# Simultaneous Optimization and Integration of Multiple Process Heat Cascade and Site Utility Selection for the Design of a New Generation of Sugarcane Biorefinery

**DOI:** 10.3390/e26060501

**Published:** 2024-06-08

**Authors:** Victor Fernandes Garcia, Adriano Viana Ensinas

**Affiliations:** 1 Center of Engineering, Modeling and Social Science Applied, Federal University of ABC, Santo André 09210-580, Brazil; v.garcia@ufabc.edu.br; 2 Department of Engineering, Federal University of Lavras, Lavras 37000-200, Brazil

**Keywords:** biorefinery, MILP superstructure, carbon credit, process integration, biofuels, optimization, heat integration

## Abstract

Biorefinery plays a crucial role in the decarbonization of the current economic model, but its high investments and costs make its products less competitive. Identifying the best technological route to maximize operational synergies is crucial for its viability. This study presents a new superstructure model based on mixed integer linear programming to identify an ideal biorefinery configuration. The proposed formulation considers the selection and process scale adjustment, utility selection, and heat integration by heat cascade integration from different processes. The formulation is tested by a study where the impact of new technologies on energy efficiency and the total annualized cost of a sugarcane biorefinery is evaluated. As a result, the energy efficiency of biorefinery increased from 50.25% to 74.5% with methanol production through bagasse gasification, mainly due to its high heat availability that can be transferred to the distillery, which made it possible to shift the bagasse flow from the cogeneration to gasification process. Additionally, the production of DME yields outcomes comparable to methanol production. However, CO_2_ hydrogenation negatively impacts profitability and energy efficiency due to the significant consumption and electricity cost. Nonetheless, it is advantageous for surface power density as it increases biofuel production without expanding the biomass area.

## 1. Introduction

The economic development of a region is linked to the increase in its energy consumption. At present, the world’s energy matrix is mainly composed of non-renewable sources; therefore, in a global scenario of worsening climate change, the development of renewable energy sources that do not cause greater greenhouses gas emissions is fundamental for sustainable development and the creation of a low-carbon economy. In this sense, biorefineries play a crucial role in sustainable economic development by enabling the recovery of waste that would otherwise be discarded. A biorefinery is a collection of processes that can sustainably convert biomass into marketable products such as bioplastics, biofuels, and chemical intermediates. Several biorefineries have been developed and implemented, including those for sugarcane, wood, microalgae, and municipal solid waste. However, the presence of biofuels and other products from renewable resources is still low due to technological and economic barriers. To achieve competitive improvement, a biorefinery must operate in an optimized manner, making sustainable use of all available resources. However, the diversity of resources, processes, and products makes it difficult to identify this optimal configuration, making the development of a biorefinery a complex and difficult task to solve. In this sense, superstructure modeling and optimization, a computational tool used to generate and evaluate systematically all the configurations that element sets can present, have been successfully used in process synthesis. This approach uses mathematical programming to identify the ideal combination of a set of alternatives that should be adopted to achieve a given objective. In process synthesis engineering, the superstructure is widely used in heat exchange network synthesis, conversion route evaluation, and supply chain networks. A superstructure typically consists of an objective function used to evaluate and compare different outcomes, along with a set of constraints, variables, and parameters. Optimization by superstructure is a viable solution to this problem, as evidenced by the results of several researchers, as will be presented. Based on thermodynamic laws, process heat integration (HI) combined with pinch analysis (PA) is an essential tool that can increase the economic viability of a biorefinery while reducing its carbon dioxide (CO_2_) emissions from the utility system [1]. This method identifies opportunities for heat exchange between heat flows within the same or different processes, thereby reducing fuel consumption in the utility system. When the method is applied considering different processes, it is known as total site heat integration (TSHI) [2]. Various works in the literature apply TSHI concepts to superstructures [3,4,5,6]. Due to the combinatorial nature of the problem, most studies implement sequential strategies to solve it. However, this approach lacks the guarantee of finding a global optimal solution and may overlook more suitable options for the specific problem. This approach can have a high computational cost, making its application impractical in certain cases. Limitations are also present in other works, as they offer specific formulations for certain problems.

The sugar and alcohol sector is a crucial industry for the Brazilian economy, with Brazil currently ranking second in the world in bioethanol production. Traditional bioethanol production in the sugarcane industry is a consolidated process in which bioethanol is produced from the sugars present in sugarcane juice in seven stages: cleaning, preparation, and extraction; processing; concentration; sterilization and cooling; fermentation; distillation; and dehydration. First, the sugarcane arrives at the distillery, where it undergoes a process of cleaning, cutting, and then grinding. The extracted juice is sent to the treatment stage, where impurities are removed through a coagulation and decantation process. The bagasse produced is used as fuel in the utility system. Next, the treated broth is sent to an evaporator system where it is concentrated by an evaporator system until it reaches 19°Brix (a unit of measurement used in the industry to express the mass of sugars in a solution) [7]. In the sterilization stage, the already concentrated broth is heated to 130 °C and then cooled to 32 °C. During fermentation, the sugars present in the broth are consumed and converted into bioethanol by the action of yeasts of the genus Saccharomyces cerevisiae. It also produces carbon dioxide, which is released into the atmosphere after purification. To recover the bioethanol, the wine produced is sent to a distillation column system made up of four columns (A, A1, D, and B-B1). At this stage of the process, hydrated bioethanol (92.6–93.8% by mass of bioethanol) and a stream of vinasse [8] are obtained, a dark brown effluent, acidic in nature and with a high pollution potential. For each liter of bioethanol, 10 to 15 L of vinasse are produced [9]. To achieve a concentration of 99.3% by mass for bioethanol, the hydrated bioethanol undergoes a dehydration process in which excess water is removed using a solvent such as monoethylene glycol (MEG) or cyclohexane [10].

A standard distillery uses a cogeneration system to meet its energy needs, generating both electrical and thermal energy for all stages of the process. This system consists of a boiler, steam turbine, and electrical generator that form a steam cycle. Bagasse is used as a fuel to heat water and produce superheated steam. The resulting steam is sent to the steam turbine to expand and generate electricity. The steam then moves to the process, provides the required energy, and finally returns to the cogeneration system. In certain cases, there may be an excess of steam, which is then transported to thermoelectric facilities. The excess electricity produced by these plants is then sold to the power grid. According to Albarelli [10], improvements in energy integration of the process with investments in heat recovery technology can make a large amount of bagasse available as feedstock for other processes, such as gasification integrated with methanol production. According to Fuess et al. [11], the integration of new processes, in addition to increasing energy efficiency, would diversify the products obtained, improving the biorefinery nature of the sugarcane industry. In this sense, the biodigestion of vinasse is a possibility to be evaluated. It is usually used for the fertigation of sugarcane crops, but its polluting properties limit its use in the soil. In addition, the large quantity produced makes its proper disposal a problem for the distillery. Thus, biodigestion of vinasse, in addition to reducing its pollution potential, would produce biomethane, a biofuel considered strategic for the energy transition [12]. Methanol is a product that can be blended with gasoline, is used in the production of biodiesel or directly in fuel cells, and is commonly obtained from natural gas; the conversion of carbon dioxide to methanol is a process that has received attention [13,14].

As mentioned earlier, determining the configuration of a biorefinery can be a complex task due to the numerous processes that can be integrated. To optimize biorefineries and identify the best production route, several authors have adopted the use of superstructure as an alternative. Infante et al. [15] presented a MILP formulation to evaluate a microalgae biorefinery considering the production of different biofuels in Colombia. Its formulation found that microalgae liquefaction was the most viable route, while bagasse was used as process fuel and pellet production. Fonseca et al. [16] formulated a superstructure to evaluate the best strategy and the economic feasibility of integrating a second-generation ethanol process into an existing distillery. The results suggested that all bagasse was allocated to hydrolysis, while sugarcane straw, lignin, and biogas were directed to a Rankine Cycle. Huynh et al. [17] used a superstructure aiming to maximize the biodiesel production profit. The study by Kenkel et al. [18] employs a bicriteria superstructure, a superstructure with two objective functions, to investigate the conversion of CO_2_ to methanol in Germany. The authors found that the price of electricity significantly influences the selection of technologies, impacting directly the production costs and emissions. The study suggests that synthetic methanol production from renewable energy sources could become competitive with natural gas in the future if its cost were reduced. Pyrgakis and Kokossis [19] employed a superstructure with a bipartite graphical representation and a modified total site cascade to study a real lignocellulosic biorefinery. The proposed formulation identified operational synergies between the thermal currents of the processes, which reduced the demand for hot and cold utilities by 9% and 14%, respectively. Celebi et al. [20] proposed a multiobjective superstructure for comparing sugar and syngas biorefinery platforms, ranking thirty-four configurations, with the lowest cost configuration integrating DME production with succinic acid. With its superstructure formulation, Galanopoulos et al. [21] reduce the biodiesel production costs by up to 80% in an integrated algae biorefinery using wastewater and CO_2_ emissions. As highlighted above, the approach taken by the works considers sequential procedures to solve problems, so it lacks the guarantee of finding a globally optimal solution and may ignore more appropriate options for the specific problem. To address this issue, this paper presents a new superstructure formulation that utilizes mixed integer linear programming (MILP) for biorefinery optimization. The presented model’s innovation lies in its ability to perform the process selection with scale adjustment, simultaneously to utility selection, and heat recovery by heat cascade integration. Unlike other approaches, this method results in the optimal configuration of the biorefinery, ensuring that the presented configuration is the best possible.

## 2. Methodology

**Figure 1:**
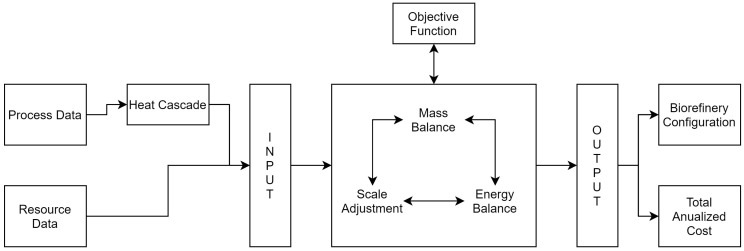
Schematic representation of superstructure flow information.

The formulation presented in this paper consists of general mass and energy balance and is based on the previous work of Kantor et al. [22], while the constraints to perform heat cascade constraint is based on Bagajewicz and Rodera [23]. To achieve this, black box models representing each technology are inserted into the biorefinery. Each of the inserted models is based on previous works and describes the conversion, input, and output flows of each process, as well as their thermal stream, which are used to perform the heat cascade integration. Therefore, the superstructure receives information related to process and resource economics, including process operation, maintenance, and investment costs, as well as resource acquisition costs and market prices. The superstructure was modeled as a MILP model and implemented in LINGO software v.21 [24]. Figure 1 shows the flow of information in the superstructure. Next, the formulation of the superstructure is presented.

### 2.1. Main Sets Definitions

**Figure 2:**
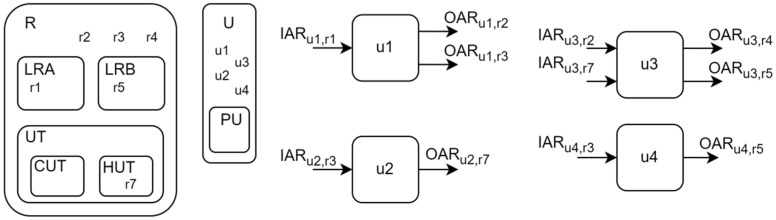
Superstructure main sets and subsets representation, and the inlets and outlets units.

The formulation considers different sets, subsets, and their combinations. This structure makes it possible to perform the inclusion and exclusion of processes and their parameters in a faster and more organized way, in addition to allowing the superstructure to be extended by including new concepts in sets. There are two main sets, RESOURCE (R) and UNIT (U). An r element (r ∈ R) represents anything that can be transported, e.g., a biomass or a utility, while a u element (u ∈ U) represents a unit that can transform one resource into another, e.g., a distillery that transforms sugarcane into bioethanol (EtOH), vinasse, bagasse, and CO_2_. Each unit is inserted into the superstructure as a black box model. In the PROCESS (PU) subset (PU⊂U), the elements represent units that have at least one heat stream available for heat integration. UTILITIES (UT) subset (UT⊂R) contains the elements that represent thermal utilities, while HUT (HUT⊂UT) and CUT (CUT⊂UT) contain the hot and cold utilities, respectively. The elements in the sets LRA (LRA⊂R) and LRB (LRB⊂R) are the resources that can be acquired and commercialized. Figure 2 shows a schematic representation of the main sets and subsets in the superstructure.

### 2.2. Objective Function

As an objective function presented in Equation (1), the superstructure considers the minimization of the total annual cost (TAC), which considers the annualized unit capital cost (UCCu), the resource acquisition cost (RCr), the product commercialization revenue (PCr), and the carbon credits (CCs) resulting from the replacement of fossil fuels by their respective renewable energy sources, as expressed in Equation (1).

(1) $$\mathrm{TAC}=\sum_{u}{\mathrm{UCC}}_{u}+\sum_{r}{\mathrm{RC}}_{r}-\sum_{r}{\mathrm{PC}}_{r}-\;\mathrm{CC}$$

### 2.3. Unit Selection and Scale Adjustment

The selection and scale adjustment of each element *u*, is carried out by Equation (2), where ${\mathrm{CapMin}}_{u}$ and ${\mathrm{CapMax}}_{u}$ are parameters that represent the maximum and minimum scale adjustment that a unit can have, $y_{u}$ is a binary variable that represents the existence of that unit, and $w_{u}$ is a continuous variable responsible for the unit scale adjustment. When selected, a unit has its $y_{u}$ equal to one, and its $w_{u}$ is limited by ${CapMin}_{u}$ and ${CapMax}_{u}$. If not selected, $y_{u}$ assume value zero, resulting in a $w_{u}$ equal to zero.

(2) $${\mathrm{CapMin}}_{u}y_{u}\leq w_{u}\leq {\mathrm{CapMax}}_{u}y_{u}\;\;\;\;\;\forall u\in U,\forall l\in L$$

### 2.4. Mass Balance

As mentioned above, the superstructure uses the concept of scale adjustment. In this concept, each element *u* is inserted as a black box model with a specific scale and its input flow $\left({\mathrm{IAR}}_{u,r}\right)$ and output flow $\left({\mathrm{OAR}}_{u,r}\right)$ for each resource, and it is relative to the specific scale. Depending on the situation, the scale of this unit can be adjusted, increased, or decreased. To do this, a continuous variable $w_{u}$ is multiplied by each one of these parameters, adjusting them linearly and proportionally to the required scale. Thus, the produced and consumed quantities of a resource are calculated by Equations (3) and (4) respectively, where ${prod}_{u,r}$ is the production of resource *r* by unit *u* and ${cons}_{u,r}$ is the consumption of resource *r* by unit *u*.

(3) $${\mathrm{OAR}}_{u,r}w_{u}-{\mathrm{prod}}_{u,r}=0\;\;\;\;\;\forall u\in U,\forall r\in R$$

(4) $${\mathrm{IAR}}_{u,r}w_{u}-{\mathrm{cons}}_{u,r}=0\;\;\;\;\;\forall u\in U,\;\forall r\in (R-\mathrm{UT})$$

As the superstructure does not consider the accumulation of resources, every resource produced or bought needs to be consumed or sold. To represent this condition, Equations (5)–(7) were developed, where bought_r_, sold_r_, and fop represents the amount bought and sold of a resource r, and the fop is the hours of operation in a year. Equations (5) and (6) are applied to the features contained in sets LRA and LRB, respectively. Equation (7) is applied to features that are not present in the LRA and LRB subsets.

(5) $${\mathrm{bought}}_{r}=\sum_{u}{\mathrm{cons}}_{u,r}\mathrm{fop}\;\;\;\;\;\forall r\;\in \;\mathrm{LRA}$$

(6) $$\sum_{u}{\mathrm{prod}}_{u,r}\mathrm{fop}=\sum_{u}{\mathrm{cons}}_{u,r}\mathrm{fop}+{\mathrm{sold}}_{r}\;\;\;\;\;\forall r\;\in \;\mathrm{LRB}$$

(7) $$\sum_{u}{\mathrm{prod}}_{u,r}\mathrm{fop}=\sum_{u}{\mathrm{cons}}_{u,r}\mathrm{fop}\;\;\;\;\;\forall r\;\in \;R-(\mathrm{LRA}+\mathrm{LRB})$$

The Equations (8) and (9), where avail_r_ and demand_r_ represents the amount avail and demanded of resource *r*, ensure that every resource bought is available, and every resource sold is demanded.

(8) $${\mathrm{bought}}_{r}\;\leq \;{\mathrm{avail}}_{r}\;\;\;\;\;\forall r\;\in \;\mathrm{LRA}$$

(9) $${\mathrm{sold}}_{r}\;\leq \;{\mathrm{demand}}_{r}\;\;\;\;\;\forall r\;\in \;\mathrm{LRB}$$

As the superstructure enables the selection of utilities simultaneously with total site heat integration, the consumption of a thermal utility (element contained in UT) by a process (element contained in PU) cannot be defined as a parameter, as it can vary depending on the scale of the process and whether it is energetically integrated with other processes. Therefore, for a pu element, Equation (4) is rewritten as Equation (10), where ${massUtility}_{pu,ut}$ is the value of the mass flow of utility ut consumed by the pu process.

(10) $${\mathrm{massUtility}}_{\mathrm{pu},\mathrm{ut}}-{\mathrm{cons}}_{\mathrm{pu},r}=0\;\;\;\;\;\forall \mathrm{pu}\;\in \;\mathrm{PU},\;\forall r\;\in \;\mathrm{UT}$$

### 2.5. Multiple Cascade Heat Integration and Utility Selection

The energy balance and energy integration constraints between cascades were used and applied to all *pu* elements to perform utility selection and energy integration. These constraints identify regions of heat exchange between processes, as well as between processes and utilities. For this, it is necessary that the superstructure receives the heat cascade formulation, that is, the number of stages (s) and the inlet $\left({\mathrm{Te}}_{s}\right)$ and outlet $\left({\mathrm{Ts}}_{s}\right)$ temperature of each one, the minimum amount of energy required $({\mathrm{MER}}_{\mathrm{pu}}),$ and the minimum consumption of cold utility $\left({\mathrm{UF}}_{\mathrm{pu}}\right)$ of each *pu* element, in addition to the inlet $\left({\mathrm{Tin}}_{\mathrm{pu},n}\right)$ and outlet $\left({\mathrm{Tout}}_{\mathrm{pu},n}\right)$ temperature for each stream and its respective thermal capacity (${\mathrm{MCp}}_{\mathrm{pu},n})$.

Equations (11) and (12) were used to perform the selection of utilities and heat integration simultaneously, which relate the minimum amount of energy that a process needs to receive/transfer with its possible sources/receivers. Equation (11) expresses that the heat demanded by a process is equal to the heat received in the form of utilities (${\mathrm{Qu}}_{\mathrm{pu},\mathrm{ut}}$) or by direct integration with another process (${\mathrm{Qin}}_{\mathrm{pu}}$). Since it is possible that there is more than one utility that can supply heat, the heat received from utilities is placed is special summations that limits the utilities that can exchange heat. Equation (12) express that the cold utility consumed by a process is equal to the heat transferred to a cold utility ut (${\mathrm{Qu}}_{\mathrm{pu},\mathrm{ut}}$) or transferred to another process (Qout_pu_).

(11) $${\mathrm{MER}}_{\mathrm{pu}}w_{\mathrm{pu}}=\sum_{\mathrm{ut}}{\mathrm{Qu}}_{\mathrm{pu},\mathrm{ut}}+{\mathrm{Qin}}_{\mathrm{pu}}\;\;\;\;\;\forall u\in \mathrm{PU},\;\forall \left(\mathrm{ut}\;\in \;\mathrm{HUT}\;\wedge \;{\mathrm{UTout}}_{\mathrm{ut}}\;\geq \;{\mathrm{Tpinch}}_{\mathrm{ut}}\right)$$

(12) $${\mathrm{UF}}_{\mathrm{pu}}w_{\mathrm{pu}}=\sum_{\mathrm{ut}}{\mathrm{Qu}}_{\mathrm{pu},\mathrm{ut}}+{\mathrm{Qout}}_{\mathrm{pu}}\;\;\;\;\;\forall u\in \mathrm{PU},\;\forall \left(\mathrm{ut}\;\in \;\mathrm{CUT}\;\wedge \;{\mathrm{UTout}}_{\mathrm{ut}}\;\leq \;{\mathrm{Tpinch}}_{\mathrm{ut}}\right)$$

**Figure 3:**
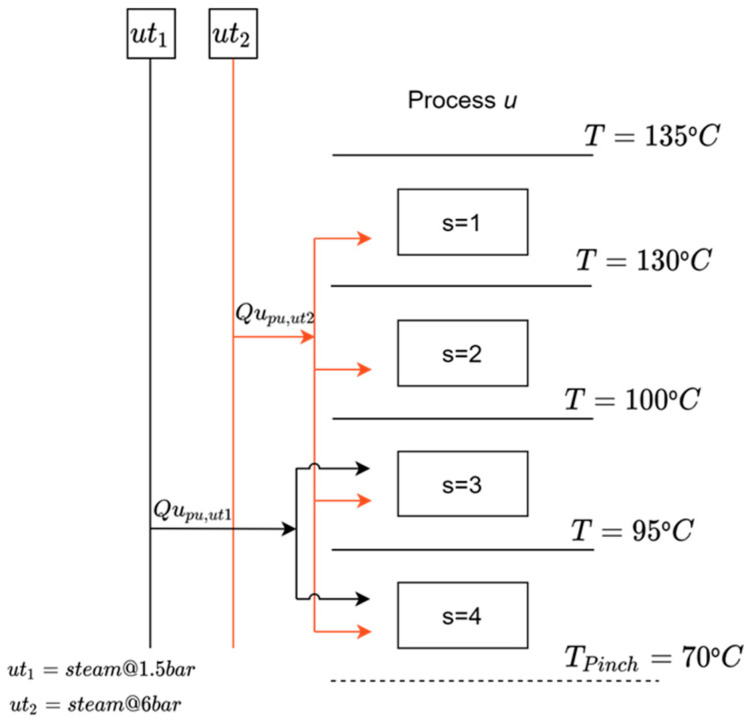
Representation of the utility placement in heat cascade.

When a unit is scaled, its heat demand must be adjusted proportionally as its scale increases or decreases. This is done by multiplying its minimum energy requirement of hot utility (MER_pu_) and cold utility (UF_pu_) by its scaling variable (w_pu_). The heat supplied by a utility to a unit is determined by the unit pinch temperature (Tpinch_pu_) Equations (13)–(15), where Qh_pu,s_ is the heat available by the hot streams in stage *s* by process *pu* and Qc_pu,s_ is the heat demanded by the cold streams in stage s by process pu and is limited by the interval temperature of the heat cascade stage and the heat demand of that stage. Figure 3 shows a representation of the utility placement in heat cascade as a general example.

(13) $${\mathrm{Qu}}_{\mathrm{pu},\mathrm{ut}}\leq \sum_{s|\;{\mathrm{Ts}}_{s}\geq \;{\mathrm{Tpinch}}_{\mathrm{pu}}\;\wedge \;{\mathrm{Te}}_{s}\leq {\mathrm{UTout}}_{\mathrm{ut}}}\left({\mathrm{Qh}}_{\mathrm{pu},s}-{\mathrm{Qc}}_{\mathrm{pu},s}\right)w_{\mathrm{pu}}\;\;\;\;\;\forall \mathrm{puPU},\;\forall \;\mathrm{ut}\;\in \;\mathrm{HUT}$$

(14) $${\mathrm{Qh}}_{\mathrm{pu},s}=\sum_{n|\;{\mathrm{Tin}}_{\mathrm{pu},n}\geq \;{\mathrm{Te}}_{s}\;\wedge \;{\mathrm{Tout}}_{\mathrm{pu},n}\leq {\mathrm{Ts}}_{s}}{\mathrm{MCp}}_{\mathrm{pu},n}\left({\mathrm{Te}}_{s}-{\mathrm{Ts}}_{s}\right)\;\;\;\;\;\forall \mathrm{pu}\;\in \;\mathrm{PU};\;\forall s$$

(15) $${\mathrm{Qc}}_{\mathrm{pu},s}=\sum_{n|\;{\mathrm{Te}}_{s}\leq \;{\mathrm{Tout}}_{\mathrm{pu},n}\;\wedge \;{\mathrm{Ts}}_{s}\geq {\mathrm{Tin}}_{\mathrm{pu},n}}{\mathrm{MCp}}_{\mathrm{pu},n}\left({\mathrm{Te}}_{s}-{\mathrm{Ts}}_{s}\right)\;\;\;\;\;\forall \mathrm{pu}\;\in \;\mathrm{PU};\;\forall s$$

Equations (16) and (17) are responsible for converting the heat demand of a utility into its mass flow, connecting mass balance and energy balance.

(16) $${\mathrm{Qu}}_{\mathrm{pu},\mathrm{ut}}={\mathrm{massUtility}}_{\mathrm{pu},\mathrm{ut}}{\mathrm{hv}}_{\mathrm{ut}}\;\;\;\;\;\forall u\;\in \;\mathrm{PU},\;\forall \mathrm{ut}\;\in \;\mathrm{HUT}$$

(17) $${\mathrm{Qu}}_{\mathrm{pu},\mathrm{ut}}={\mathrm{massUtility}}_{\mathrm{pu},\mathrm{ut}}{\mathrm{hs}}_{\mathrm{ut}}\;\;\;\;\;\forall u\;\in \;\mathrm{PU},\;\forall \mathrm{ut}\;\in \;\mathrm{CUT}$$

To consider the heat entering and leaving one HC stage to another HC, the variables Qf and Qs have been inserted, and this represents the inlet heat into unit pu and stage s and Outlet heat into unit pu and stage s, respectively. For the stages below the pinch temperature, Qf has a value of zero, while for the stages above the pinch, Qs has a value of zero. These considerations are made to ensure that there is no heat input into the region below the pinch or heat loss above the pinch. This consideration is expressed in Equations (18) and (19), as well as the limitation of the amount of heat that can enter or leave a stage of the thermal cascade.

(18) $${\mathrm{Qf}}_{\mathrm{pu},s}\left\{\begin{array}{c} =0\\ \leq \left({\mathrm{Qc}}_{\mathrm{pu},s}-{\mathrm{Qh}}_{\mathrm{pu},s}\right)w_{\mathrm{pu}} \end{array}\right.$$

(19) $${\mathrm{Qs}}_{\mathrm{pu},s}\left\{\begin{array}{c} =0\\ \leq \left({\mathrm{Qh}}_{\mathrm{pu},s}-{\mathrm{Qc}}_{\mathrm{pu},s}\right)w_{\mathrm{pu}} \end{array}\right.$$

**Figure 4:**
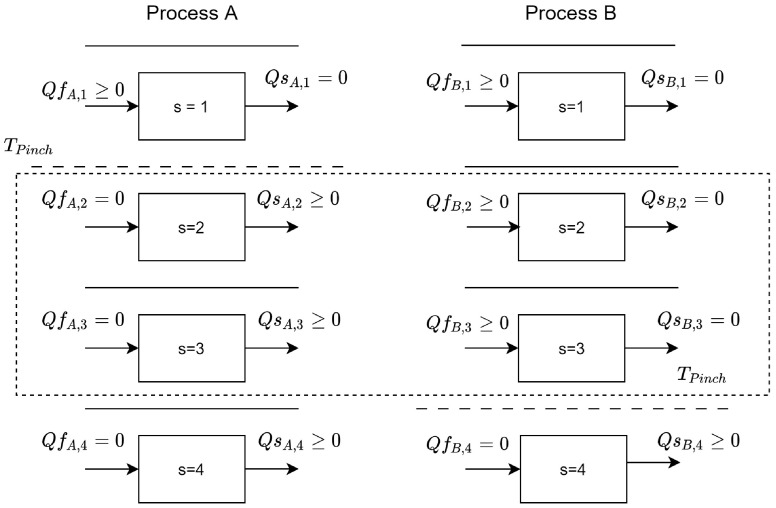
Region between pinch process, which allows heat integration.

By preventing heat from leaving the region above the pinch or entering the region below the pinch of an HC, heat transfer from one HC to another HC is limited to the region between the pinches of the two HCs, with heat leaving the one with the higher pinch temperature and entering the lowest pinch temperature. This region is shown in Figure 4.

The total amount of heat transferred from one process to another is calculated by Equation (20). Equation (21) expresses the total heat received from other processes. Equation (22) limits the heat released by a process to the total heat received by others in the region bounded by the pinch. Equation (23) states that the heat received by a unit must be less than or equal to the total heat transferred by the others in the region between the pinches.

(20) $${\mathrm{Qout}}_{\mathrm{pu}}=\sum_{s|\;{\mathrm{Te}}_{s}\leq \;{\mathrm{Tpinch}}_{\mathrm{pu}}}{\mathrm{Qs}}_{\mathrm{pu},s}$$

(21) $${\mathrm{Qin}}_{\mathrm{pu}}=\sum_{s|\;{\mathrm{Ts}}_{s}\geq \;{\mathrm{Tpinch}}_{\mathrm{pu}}}{\mathrm{Qf}}_{\mathrm{pu},s}$$

(22) $${\mathrm{Qout}}_{\mathrm{pu}}\;\leq \;\sum_{{pu}^{\prime }|\;{\mathrm{Tpinch}}_{{\mathrm{pu}}^{\prime }}>\;{\mathrm{Tpinch}}_{\mathrm{pu}}}\sum_{S|\;{\mathrm{Te}}_{s}\leq {\mathrm{Tpinch}}_{pu}\&{\mathrm{Ts}}_{s}\geq {\mathrm{Tpinch}}_{{\mathrm{pu}}^{\prime }}}{\mathrm{Qf}}_{{\mathrm{pu}}^{\prime },s}$$

(23) $${\mathrm{Qin}}_{\mathrm{pu}}\;\leq \;\sum_{{\mathrm{pu}}^{\prime }|\;{\mathrm{Tpinch}}_{pu^{\prime }}<\;{\mathrm{Tpinch}}_{\mathrm{pu}}}\sum_{{\mathrm{Te}}_{s}\leq {\mathrm{Tpinch}}_{{\mathrm{pu}}^{\prime }}\&{\mathrm{Ts}}_{s}\geq {\mathrm{Tpinch}}_{pu}}{\mathrm{Qs}}_{{\mathrm{pu}}^{\prime },s}$$

Equation (24) guarantees that the total heat output of the processes is equal to the total heat input of the processes.

(24) $$\sum_{\mathrm{pu}}{\mathrm{Qin}}_{\mathrm{pu}}=\sum_{\mathrm{pu}}{\mathrm{Qout}}_{\mathrm{pu}}$$

### 2.6. Unit Capital Cost and Investment Cost Linearization

**Figure 5:**
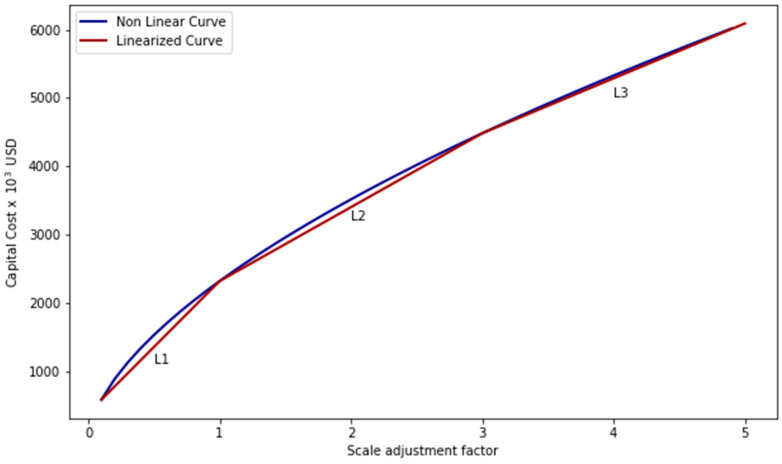
Piecewise linearization of the investment cost function of a process.

Since each unit inserted in the superstructure has a reference scale, in addition to the resource consumption and production values, each of them has a capital cost related to the scale considered. Process capital costs tend to vary nonlinearly with scale, so to maintain the model linearity, a piecewise linearization of the capital cost function was performed for each process. First, Equation (25) was used to obtain the cost curve as a function of the scaling factor (w_u_), where $C_{u}$ is the adjusted capital cost for unit u, ${C0}_{u}$ is the annualized capital cost at the reference scale for unit u, and se is a scaling exponent. As recommended by Peters et al. [25] it was considered that the unit *u* could be reduced or increased by up to 10 times the reference value. In this way, the curve obtained starts with 10% of the unit’s reference value and ends with 1000% of the unit’s reference value. The curve is then divided into three levels limited by a minimum and maximum value, ${\mathrm{CapMin}}_{u,l}$ and ${\mathrm{CapMax}}_{u,l}$, respectively, as shown in Figure 5. The linearized cost curve coefficients for each process are present in the Supplementary Materials.

(25) $$C_{u}={\;C0}_{u}w_{u}^{\mathrm{se}}$$

When a unit is selected and scaled, one of the levels must also be selected. Therefore, Equation (2) is rewritten as Equation (26). Since only one level can be selected when a unit is selected, Equation (27) guarantees that only one level is selected. If a level is not selected, its binary variables (yl_u,l_) will have a value of zero, so the local scaling factor variable ${\mathrm{wl}}_{u,l}$ will also have a value of zero, so Equation (28) guarantees that $w_{u}$ will be equal to the value of ${\mathrm{wl}}_{u,l}$ of the selected level.

(26) $${\mathrm{CapMin}}_{u,l}y_{u,l}\;\leq \;{\mathrm{wl}}_{u,l}\;\leq \;{\mathrm{CapMax}}_{u,l}y_{u,l}$$

(27) $$\sum_{l}{\mathrm{yl}}_{u,l}\;\leq \;1\;$$

(28) $$\sum_{l}{\mathrm{wl}}_{u,l}=w_{u}$$

The capital cost of a unit can be determined by Equation (29), where $a_{u,l}$ and $b_{u,l}$ are the angular and linear coefficients of each linearized segment, respectively, MC, OC, AF and LC are maintenance cost, operation cost, annualization factor, and other cost, which are fixed as 6%, 8.6%, 0.086, and 10%, respectively. Equations (30)–(32) calculate the resource acquisition cost, product commercialization revenue, and carbon credits revenue, respectively, where ResCost_r_ is the cost of resource r, MPr is the market price of resource r, PC_r_ is the commercialization revenue of resource r, and CarbVal is the carbon credit price.

(29) $${\mathrm{UCC}}_{u}=\sum_{l}(a_{u,l}{\mathrm{wl}}_{u,l}+b_{u,l}y_{u,l}).\mathrm{AF}.(1+\mathrm{MC}+\mathrm{OC}+\mathrm{LC})$$

(30) $${\mathrm{RC}}_{r}={\mathrm{Bought}}_{r}{\mathrm{ResCost}}_{r}$$

(31) $${\mathrm{PC}}_{r}={\mathrm{Sold}}_{r}{\mathrm{MP}}_{r}$$

(32) $$\mathrm{CC}=\left(\sum_{r}{\mathrm{Sold}}_{r}{\mathrm{MP}}_{r}\;-\sum_{r}{\mathrm{Bought}}_{r}{\mathrm{ResCost}}_{r}\right)\mathrm{CarbVal}$$

The capital cost of each process was corrected using the CEPCI index. To annualize the process, before linearization, each curve was multiplied by the annualization factor expressed by Equation (33), considering a plant lifetime (n) of 25 years for all units and an interest rate (i) of 7%.

(33) $$\mathrm{AF}=\frac{{i\left(1+i\right)}^{n}}{{\left(1+i\right)}^{n}-1}$$

## 3. Sugarcane Biorefinery Case Studies Description

**Figure 6:**
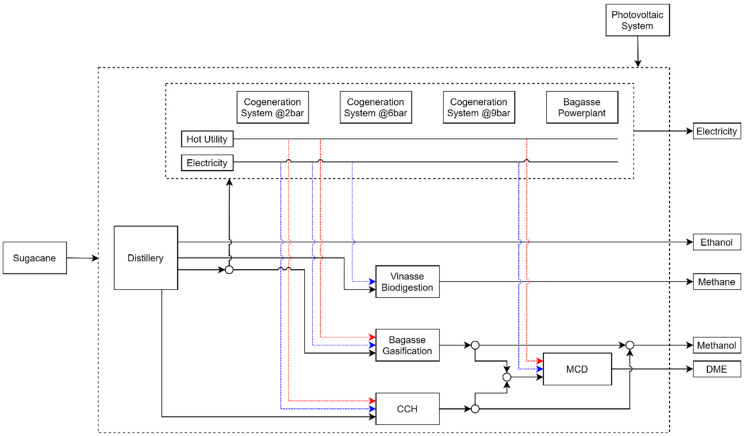
Biorefinery process superstructure.

In this paper, a conventional Brazilian sugarcane distillery with a typical processing capacity of 2,640,000 tons of sugarcane per year is considered, as previously described and performed by others authors [8]. For the proposed study, different technologies are considered to compare their impact on the performance of the biorefinery. In this study, several cases have been evaluated where different technologies have been integrated to improve the performance of the biorefinery by utilizing the wastes generated during the production of bioethanol from sugarcane juice. The wastes considered were vinasse, sugarcane bagasse, and carbon dioxide generated during the fermentation process. This study aimed to convert vinasse through the biodigestion process and bagasse through the gasification process to produce methanol or a bagasse power plant to produce and export electricity. For the carbon dioxide stream, the CO_2_ catalytic hydrogenation process was introduced, which also produces methanol. This study also included methanol catalytic dehydration (MCD) technology, which converts methanol to DME. Because the processes require electricity and consume utilities to operate, various production technologies were included. Three cogeneration systems were evaluated to produce hot utilities, each producing saturated steam at different pressures. In addition to the cogeneration systems, the possibility of importing electricity from a photovoltaic panel system was investigated. These technologies were integrated into the superstructure to collect data from existing published work. Additional information and process descriptions are provided in the following sections. Table 1 provides a summary of the technologies used for each case evaluated, and Figure 6 summarizes the combined process in the superstructure.

Four additional indicators were included to facilitate the evaluation and comparison of technologies: payback, energy efficiency, total CO_2_ avoided, and surface power density. The payback calculation considers the total investment and cash flows, considering the purchase and sale of resources, commercialization of carbon credits, and operating costs, as shown in Equation (34). For energy efficiency, Equation (35), energy input and output flows were considered in terms of resources, which were obtained based on their respective lower heating values (LHVs), as shown in Table S1. The calculation of the surface power density considers the energy produced in the form of biofuels per area of sugarcane cultivated, assuming a productivity of 76.8 tons of sugarcane/hectare. In order to calculate the total avoided CO_2_ and consequently the generation of carbon credits, the avoided CO_2_ for sugarcane [26], bioethanol [26], biomethanol [27], bioDME [26], biomethane [26], and electricity [28] are provided in the Supplementary Materials. To commercialize carbon credits, a sales price of USD 65.00 per credit was considered. The supplemental material includes the IAR and OAR values for each unit, as well as the linearized cost curves.

(34) $$\mathrm{Payback}=\frac{\mathrm{Total}\;\mathrm{Investment}}{\mathrm{Cash}\;\mathrm{flow}}$$

(35) $$\mathrm{Energy}\;\mathrm{Efficiency}=\frac{\mathrm{Output}\;\mathrm{Energy}}{\mathrm{Input}\;\mathrm{Energy}}$$

### Technologies Description

**Figure 7:**
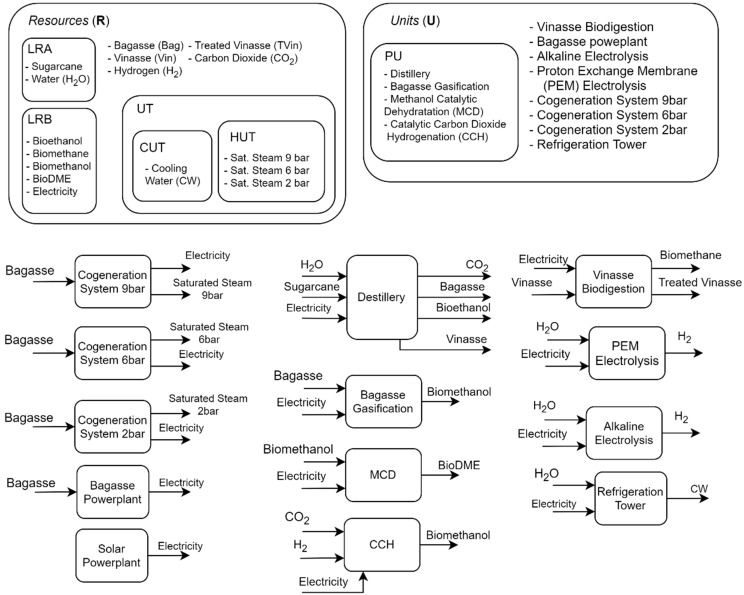
Representation of the main sets and models used in the case study.

Next, the technologies considered are described, as well as the ancillary processes for providing other resources, such as electricity and hydrogen. Figure 7 shows a representation of the superstructure formulation for this study.

**Figure 8:**
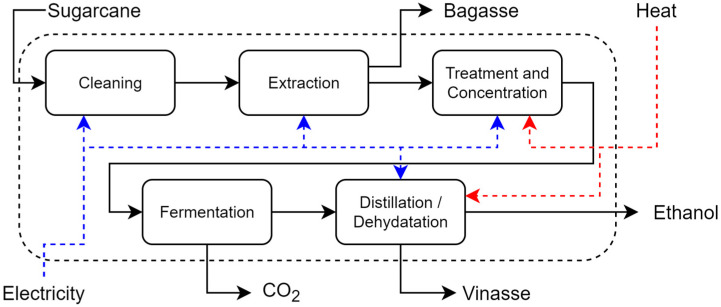
Distillery flowsheet of the sugarcane distillery.

**Distillery**: A Brazilian conventional autonomous distillery, with a typical milling capacity of 2,640,000 tons of cane per year and a crushing rate of 500 tons of sugarcane per hour is considered to produce bioethanol. This process includes the following steps: cleaning, preparation, and extraction system; cane juice treatment; juice concentration; sterilization and must cooling; fermentation; distillation and rectification; and dehydration. This unit receives sugarcane, water, electricity, and utilities as resources and produces bioethanol as the main product and bagasse, vinasse, and CO_2_ as by-products. Process and investment data were taken from [7,10]. Figure 8 shows a diagram of the sugarcane distillery.

**Figure 9:**
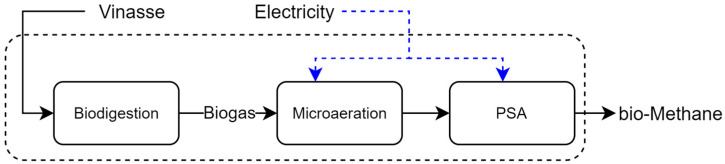
Representation of the vinasse biodigestion process.

**Vinasse Biodigestion**: The process consists of two steps, anaerobic digestion and biogas purification. In the first, the vinasse is fed directly into an anaerobic biodigester where microorganisms consume part of the organic material and produce biogas, a gas mixture of CO_2_, methane (CH_4_), and hydrogen sulfide (H_2_S). In the second step, H_2_S is removed from the produced biogas by micro-aeration of the biogas and then CO_2_ is removed by the pressure swing absorption (PSA) process, resulting in a high CH_4_ purity (>98%) [29], as shown in Figure 9.

**Figure 10:**
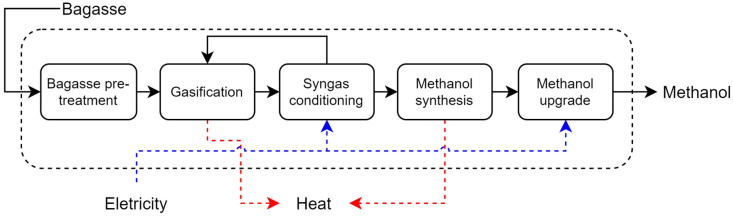
Main steps presented in bagasse sugarcane gasification integrated to methanol production.

**Bagasse Gasification**: In the first, the production of biomethanol by bagasse gasification takes place in five steps: bagasse pretreatment, gasification, syngas conditioning, methanol synthesis, and upgrading (Figure 10). In the pretreatment stage, the bagasse is dried in an air dryer and fed into the steam gasification reactor. Syngas conditioning removes major impurities such as particulates and tar from the produced gas. The composition of the syngas is adjusted with hydrogen to achieve a stoichiometric ratio s, defined by Equation (36), of 2.05, as recommended for methanol synthesis [30]. The adjusted syngas is sent to the methanol synthesis where its pressure is adjusted to 50 bar, it is mixed with unreacted syngas, and it is preheated to 225 °C before entering the reactor where its temperature is adjusted. The methanol synthesis reactor is a fixed bed reactor containing a copper/zinc oxide/alumina catalyst. The reactor effluent is decompressed and degassed. The liquid methanol is cooled to 43.3 °C and sent to a distillation column where it is purified [31]. Process and economic data were obtained from the literature [31].

(36) $$s=\frac{H_{2}-{\mathrm{CO}}_{2}}{\mathrm{CO}+{\mathrm{CO}}_{2}}$$

**Figure 11:**
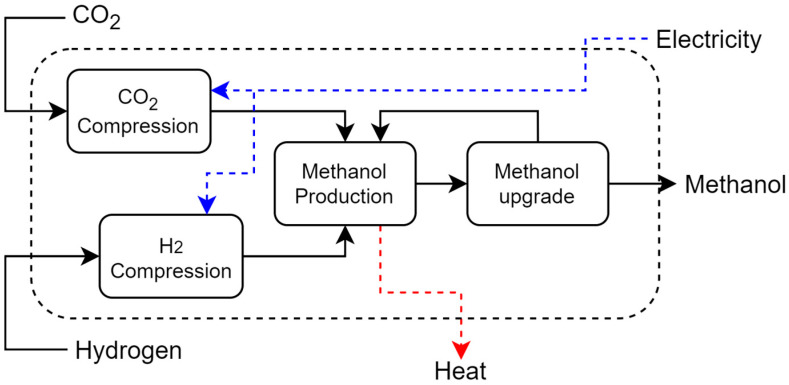
Catalytic carbon dioxide hydrogenation representation.

**Catalytic CO_2_ Hydrogenation (CCH)**: CCH is a three-step process, gas compression, methanol synthesis, and upgrading, as shown in Figure 11. First, CO_2_ is compressed to 48 bar in a 4-stage compressor and then mixed with hydrogen. Before entering the preheater, the CO_2_-H_2_ mixture receives a recycle stream of unreacted gases. The final mixture is compressed and preheated to reactor conditions (220 °C, 83 bar). The CO_2_ hydrogenation reactor is a fixed bed reactor with a Cu/ZnO/Al_2_O_3_ catalyst. The reactor effluent is cooled and decompressed; 95% of the unreacted gases are recycled and 5% is purged. The liquid methanol is cooled to 43.3 °C and fed to a distillation column as in the bagasse gasification unit. The process configuration and conditions are based on the previous work of [32,33].

**Figure 12:**
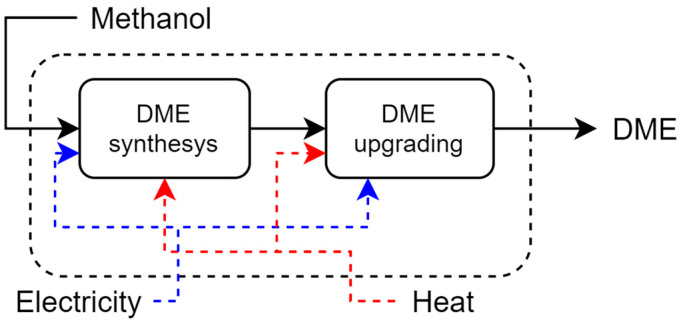
Main steps of ethanol catalytic dehydration.

**Methanol Catalytic Dehydration (MCD)**: The process can be divided into two steps, production and purification, as shown in Figure 12. In the first, fresh methanol is mixed with recycled reactant and evaporated before entering the reactor. After cooling, the effluent is sent to the purification stage where the product is recovered by column distillation. The unreacted methanol is recovered by another column and recycled. The process and economic data, including information on the process flows, were obtained by Aspen plus simulation, following the work of Shim et al. [34], Dutta et al. [31], and Turton et al. [35].

**Hydrogen Production**: Since hydrogen is required to produce methanol and DME, two different technologies are considered in the superstructure, alkaline electrolysis and proton exchange membrane electrolysis. The first is the most mature technology, and the electrodes are immersed in an aqueous KOH solution, allowing water electrolysis and thus hydrogen production. In the second, a proton exchange membrane is placed in the center of the cell to conduct the protons produced in the anode to the cathode, where they are reduced to produce hydrogen [36].

**Utilities Production**: For hot utilities, the superstructure considers three different cogeneration schemes, modeled as a steam-based cycle with steam turbines and sugarcane bagasse as fuel. Each model considers a different level of turbine output saturated steam pressure: 2.2, 6, and 9 bar. For bagasse consumption and hot utilities and electricity production, a model was developed in EES software, version 10, considering a cogeneration efficiency of 85% and a net calorific value of 7500 kJ/kg for bagasse. The operating temperatures of sugarcane ethanol distilleries are relatively low, approaching 115 °C. Consequently, the distilleries rely on cogeneration systems powered by sugarcane bagasse. To provide a more realistic representation of the process, similar systems were selected for this work. For the cold utilities, cooling water is considered [31].

**Electricity Production**: In conventional distilleries, the electricity demand is met by the cogeneration system or, in some cases, by a biomass power plant. In this sense, for electricity generation, the superstructure has three alternatives: cogeneration units (described above), bagasse, and import from a solar photovoltaic supplier.

## 4. Results and Discussion

Six different cases were evaluated after inserting the data into the superstructure. All cases considered the existence of a sugarcane distillery and the possibility of heat exchange between processes. For each of the cases, different technologies are integrated into the existing plant, making it possible to obtain a different biorefinery configuration and result. Table 2 and Table 3 provide a summary of the cost analysis results of the optimization problem solved for the cases under consideration in this paper, where the total investment represents the investment with process, resource bought represents the total expenses with resources like sugarcane. Biofuels and carbon credit revenues represent the income with biofuel and carbon credit commercialization respectively, while total avoided CO_2_ and labor cost represent the CO_2_ total mass that was no longer emitted and the cost associated with process labor cost, respectively.

**Figure 13:**
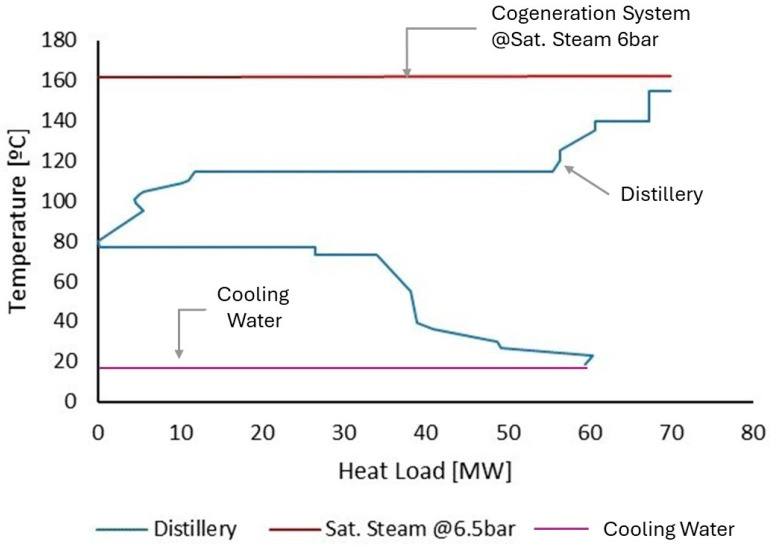
Grand composite curve with steam placement for Case 1.

For Case 1, the configuration obtained is very similar to that found in bioethanol distilleries in Brazil, which essentially consist of a distillery and a cogeneration system. The biorefinery has the potential to produce 171,072 tons of bioethanol, 729,907 tons of bagasse, 2,364,595.2 tons of vinasse (used as fertilizer), and 161,040 tons of CO_2_ per year. Figure 13 shows the initial GCC of the biorefinery with the indication of the hot utility supplied by the cogeneration system. Although the distillation column systems, from the bioethanol distillation section, are the largest consumers of utilities and require heat at a temperature close to 110 °C, the temperature of the extraction and recovery columns directly affects the selection of the utility level, causing the cogeneration system to supply heat at higher pressure levels. Thus, the cogeneration system produces saturated steam at 6.5 bar and uses 45.6 tons of bagasse per hour to produce hot utilities and electricity. Excess bagasse is diverted to a bagasse power plant, which exports excess electricity to the grid. Energetically, the biorefinery can produce 5.88 × 10^9^ MJ/year of energy with an initial energy efficiency of 50.25%.

**Figure 14:**
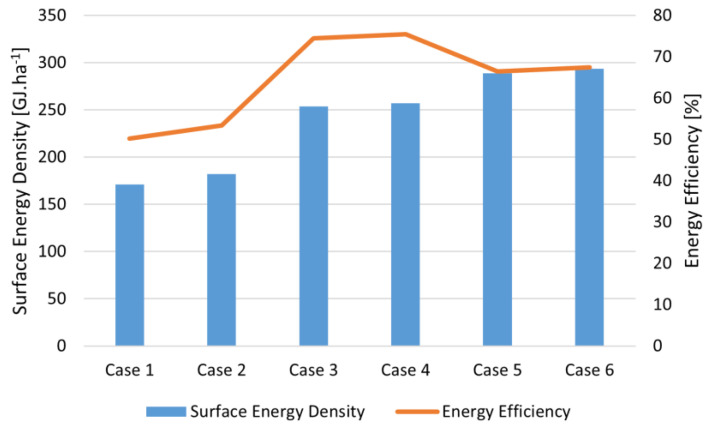
Energy efficiency comparison among all cases evaluated.

**Figure 15:**
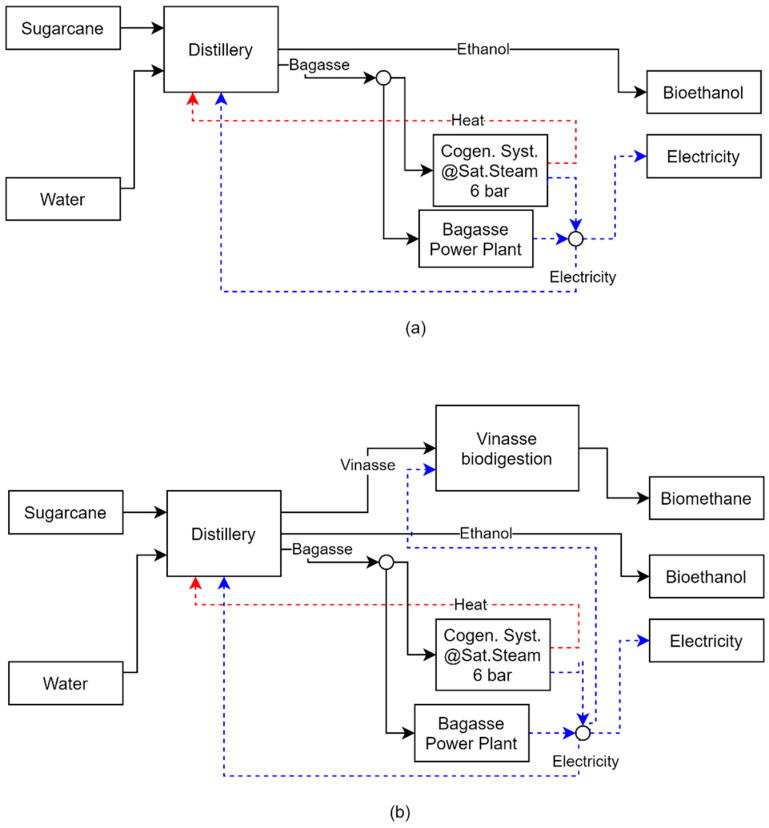
Biorefinery configuration for Case 1 (**a**) and Case 2 (**b**).

If the biodigestion of vinasse is integrated into the biorefinery, as in Case 2, the configuration remains unchanged. However, the increased consumption of the biorefinery causes a reduction in the exported electricity. The new process allows the biorefinery to produce 7075.24 tons of biomethane per year, and the biodigested vinasse is used for fermentation. Although this new unit resulted in increased TAC and payback due to higher total investment and operating costs, the production of biomethane improved environmental performance by reducing total avoided CO_2_. In addition, the increase in biofuel production from vinasse biodigestion resulted in a small increase in the energy efficiency of the biorefinery, as shown in Figure 14. In a carbon credit valorization scenario, the revenues from biomethane production can exceed the investment costs, making the process more economically viable. Figure 15a,b shows the biorefinery final configurations for Cases 1 and 2, respectively.

In Case 3, the integration of the gasification unit allowed the biorefinery to produce 209,795.6 tons of methanol per year, resulting in a 25% increase in revenue from biofuel sales. The presence of the BG unit allows heat exchange with another process, eliminating the use of utilities. The high-temperature characteristics of the BG unit result in a pinch temperature higher than that of the distillery. Therefore, when heat is exchanged, the BG unit serves as a source of thermal energy for the distillery. In addition, the heat from the BG unit was sufficient to meet the distillery’s needs. As a result, the CHP unit was not needed and was eliminated from the biorefineries’ optimal design.

**Figure 16:**
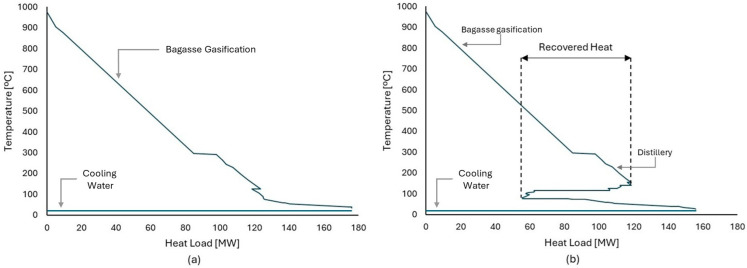
(**a**) Grand composite curve for methanol production by bagasse gasification process. (**b**) Grand composite curve for an integrated biorefinery in Case 3.

Figure 16a shows the GCC of the bagasse gasification unit. Unlike other processes, the gasification unit does not receive heat from external sources and uses a portion of the syngas produced to supply the energy required by the gasification process. As a result, two streams with a high thermal load are produced, with the first consisting of the combustion gas produced and the second consisting of the synthesis gas that has not been consumed and must be cooled before being sent to the next stages. Therefore, this unit can act as a heat source for other units present, meaning that other heat sources are not necessary. Figure 16b shows the grand composite curve of the biorefinery, and it is possible to observe that even after the integration of the distillery, there is still a large amount of heat available that can be used in other processes. It is also possible to visualize the region of the curve where it was possible to recover part of the heat present in the gasification. In this sense, when bagasse is sent to gasification, it is no longer used as fuel but instead generates revenue for the distillery while still providing heat for the processes. Thus, through energy integration, the gasification unit significantly increases the biorefinery energy efficiency, as can be observed in Figure 14.

**Figure 17:**
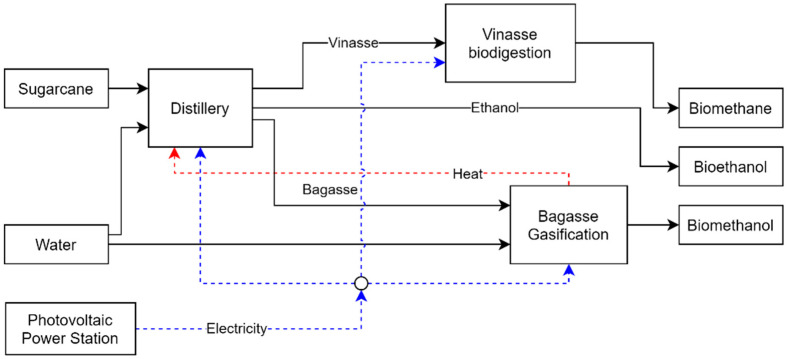
Biorefinery optimal configuration for Case 3.

Due to the delivery of the bagasse stream to the gasification unit, the biorefinery required the importation of 180,805.20 MWh of electricity from a photovoltaic system, resulting in a 37.15% increase in resource acquisition costs. Although it had a higher payback than the previous setups, Case 3 had a lower TAC, suggesting that this configuration was more beneficial over time than the others. A significant increase in this metric can be seen by examining avoided emissions. The conversion of bagasse to methanol results in the retention of more carbon in the form of biofuel, thereby increasing the amount of CO_2_ avoided. In addition, the imported electricity is supplied by a photovoltaic system, which eliminates any increase in CO_2_ emissions and results in a greater total amount of CO_2_ avoided than in the previous cases. Figure 17 shows the biorefinery configuration provided by the superstructure in this scenario.

**Figure 18:**
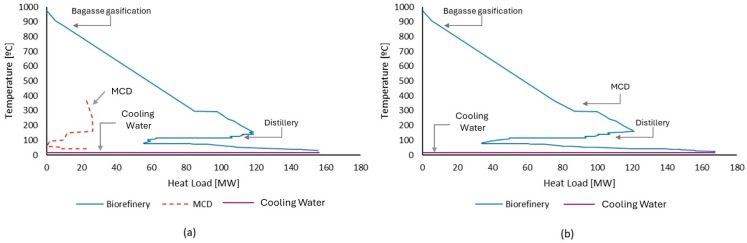
(**a**) Overlay of the biorefinery GCC (blue line) and MCD (red dotted line). (**b**) Biorefinery GCC after biorefinery integration.

In Case 4, the integration of the MCD process enabled the biorefinery to produce 148,756.60 tons of DME per year by converting all 209,795 tons of methanol produced through the bagasse gasification unit. The MCD integration required an additional investment of USD 7.94 × 10^6^ compared to Case 3. Although DME has a higher market price than methanol, the increase in operating costs and process losses led to a decrease in revenue, which negatively affected the payback of the biorefinery. The heat demand of the MCD unit can be met through energy integration with the biorefinery. Figure 18a shows the superposition of the GCC of the MCD and the biorefinery, where it is possible to observe the availability of heat that can be transferred from the biorefinery to the MCD unit. Figure 18b shows the GCC of the biorefinery after the integration of the MCD.

**Figure 19:**
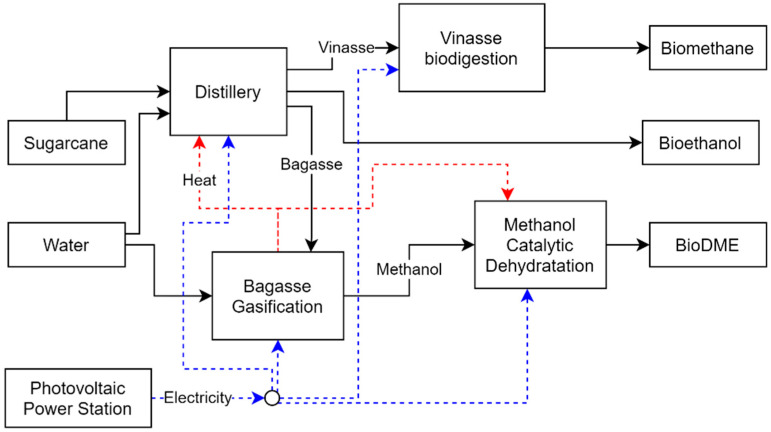
Biorefinery optimal configuration for Case 4.

Of the cases evaluated, Cases 3 and 4 presented the highest energy efficiency values. Comparing Cases 2 and 3, a significant increase in the energy efficiency of the biorefinery is observed when the gasification process is included, due to its heat transfer from gasification to the other process. Despite the higher investment required, the transfer of the bagasse flow to the gasification unit made a large amount of heat available while increasing the biofuel production, directly increasing the revenue and energy efficiency of the biorefinery. Observing Cases 3 and 4, the MCD process introduction did not have a negative impact on the energy efficiency of the biorefinery. In fact, there was a slight increase. As before, the biorefinery imported all the electricity it consumed. It received a supply of 181,759.82 MWh of electricity. Figure 19 shows the main flows in this biorefinery configuration.

**Figure 20:**
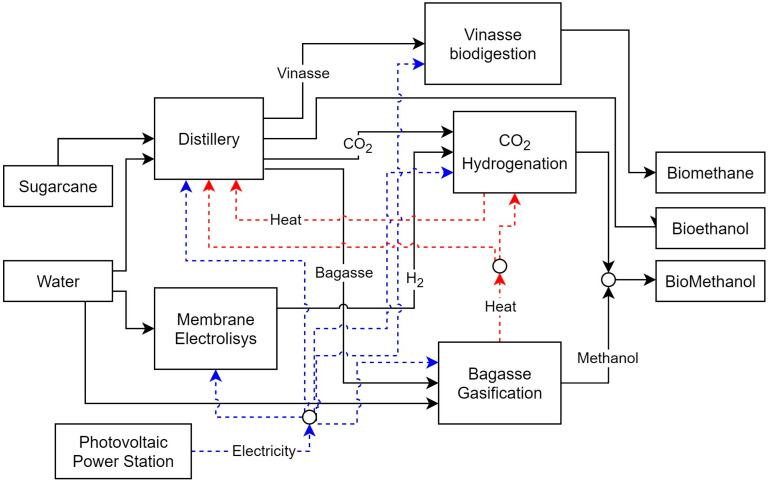
Biorefinery optimal configuration for Case 5.

In Case 5, the integration of the CCH process enabled the biorefinery to produce 306,400 tons of methanol per year, a 46% increase over Case 3. While biofuel production and carbon credit revenues increased, the payback period also increased, primarily due to the significant investment in the plant, its operating costs, and resource purchases. To convert CO_2_ into methanol, the CCH unit requires hydrogen, which must be produced by the biorefinery. Two technologies were evaluated for this purpose: alkaline electrolysis and PEM. Alkaline electrolysis was selected because of its lower capital cost. Previously, as in many distilleries, the CO_2_ produced during fermentation was vented to the atmosphere. By converting it to biofuels, the carbon capture is significantly increased, resulting in a higher total avoided CO_2_. Since the biorefinery’s CO_2_ emissions are in sugarcane cultivation and transportation, the amount of carbon credits obtained also increases. Overall, the total avoided CO_2_ increased by 21.37% compared to Case 4 and by 20.74% compared to Case 3. Figure 20 shows the biorefinery configuration and its main flows for Case 5.

**Figure 21:**
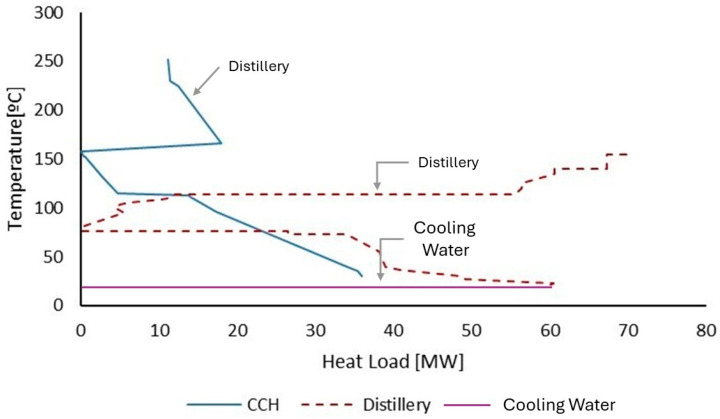
Representation of the overlap of the distillery GCC and CCH.

The CCH unit uses hot and cold utilities, allowing energy integration with other processes. By having a pinch temperature higher than that of the distillery, the CCH can transfer some of its excess heat to the distillery, as shown in the GCC of the two processes in Figure 21. At the same time, the CCH also receives heat from the gassing, thus acting as a source and sink of heat for different processes. In this sense, the biorefinery recovered 427,865 MWh of heat per year through heat integration. In Case 5, the biorefinery imported 1,403,629 MWh of electricity to power its processes. This significantly increased resource acquisition costs due to the high electricity consumption of the electrolyzer. In addition, the increased electricity consumption reduced the biorefinery’s energy efficiency to 66.46%.

As noted above, the process of converting CO_2_ into methanol using CCH requires 0.22 tons of hydrogen for each ton of methanol produced. The hydrogen must be supplied by the biorefinery. Considering all the CO_2_ conversion produced by the distillery, as well as the production of hydrogen through alkaline electrolysis, the biorefinery needed to import 1,403,629 MWh of electricity. With an energy consumption of 343.34 MWh, as hydrogen form, CCH produces 36.47 tons of methanol, resulting in an energy efficiency of 70.31% (HHV) for the CCH process. However, considering the efficiency of the electrolyzes, the overall efficiency of converting CO_2_ to methanol is 51.28%, which justifies the decrease in energy efficiency of the biorefinery. The CCH process model has a ratio of 4 moles of H_2_ to 1.13 moles of methanol, which is very close to the stoichiometric value of the reaction of 3:1. This suggests that it is essential to improve H_2_ production, electricity acquisition, or cost reduction to improve the energy efficiency of the process and the biorefinery.

**Figure 22:**
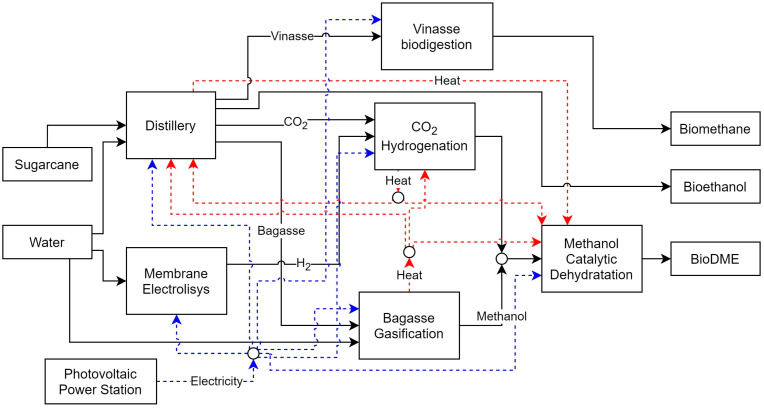
Biorefinery configuration for Case 6.

In Case 6, the biorefinery had a TAC of −31.35 × 10^6^ USD/year and required a total investment of USD 418.18 × 10^6^, resulting in an annual production of 217,260.7 tons of DME. The implementation of heat integration allowed the recovery of 603.82 GJ of energy per year. In this new configuration, the biorefinery has a 14.3% higher payback compared to the previous configuration, and a total reduction of 0.6% in avoided CO_2_ emissions. Figure 22 shows the biorefinery configuration for Case 6. Although the gasification process is present, Cases 5 and 6 show a reduction in the biorefinery’s energy efficiency. Even with heat recovery between processes, the high electricity consumption of the electrolyzers and the unavailability of resources for their production meant that the biorefinery would have to import much more electricity than in other cases, severely penalizing its energy efficiency. However, an increase in the energy produced per area of sugarcane cultivated can be observed, as in other cases where there has been an increase in biofuel production.

Comparing Cases 6 and 4, the integration of the CCH unit has led to a worsening of the economic and energy indicators, requiring more payback time to recover the investments made, and a decrease in energy efficiency. As mentioned above, the biorefinery, by producing hydrogen for the CCH, significantly increases its electricity imports and, consequently, its expenditure on this resource. This situation can also be observed when comparing Cases 3 and 5, indicating that the integration of the CCH process, despite having a strong positive impact on the generation of carbon credits, proved to be detrimental to the performance of the biorefinery. In both Cases 5 and 6, for an annual production of 21,225.6 tons of H2, the annualized cost of the electrolyzer was USD 15,437,508.52. Thus, hydrogen production has a cost of USD 4.69 per kg.

A comparison of the results obtained with other studies reveals that the values found are comparable to those reported. For a first-generation distillery, which produces ethanol from sugarcane juice, Albarelli [30] achieved an energy efficiency of close to 43%. By integrating methanol production through the gasification of bagasse and sugarcane straw, the energy efficiency of the biorefinery varied between 50% and 65%, depending on the configuration and mixture evaluated. It is crucial to emphasize that in Albarelli’s work, the authors considered a range of biorefinery configurations, including the production of second-generation ethanol utilizing a portion of straw and bagasse. Nevertheless, as in this work, the authors concluded that the increase in the energy efficiency of the biorefinery is a result of the increase in biofuel productivity. However, this is accompanied by an increase in investments and the complexity of the technologies present in the biorefinery. Bressanin [37] evaluated the production of biofuels using the Fischer–Tropsch synthesis process from two different types of sugarcane, obtaining efficiency values between 45.4% and 57.7%.

**Figure 23:**
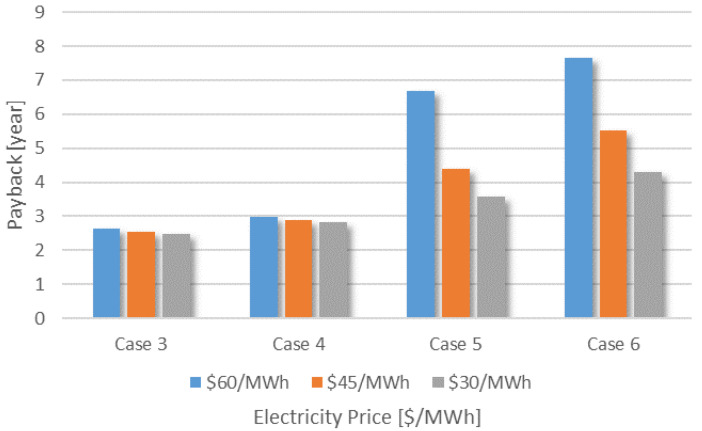
Obtained payback for different electricity prices considered.

To evaluate the impact of the electricity price on the payback of the biorefinery, Cases 3 to 6 were simulated again considering different electricity prices, 60, 45, and 30 USD·MWh^−1^. By reducing the cost of electricity, it is possible to observe a positive impact on the payback values of all cases, as shown in Figure 23. Since the electricity imports are much higher in Cases 5 and 6 than in Cases 3 and 4, the reduction of the payback time was more significant. When the cost of electricity is 45 USD·MWh^−1^, Cases 5 and 6 show a reduction of 34.8% and 27.8%, respectively. When the cost is 30 USD·MWh^−1^, the reduction is 46.5% and 43.6%, respectively, compared to the first case. These results suggest that the price of electricity is crucial to increase the competitiveness of biofuels and thus improve the viability of new generations of biorefineries as proposed in this paper. Furthermore, it is also possible to observe the impact that hydrogen production can have on the performance of a biorefinery, raising the hypothesis that the development and improvement of technologies is a point of great relevance for the development of biorefineries.

## 5. Conclusions

This study presents a novel superstructure that uses a MILP formulation to optimize and evaluate biorefineries. In this formulation, the selection and scaling of each process are performed simultaneously with the selection of utilities and heat integration between processes. The selection and scaling of processes for biorefinery composition were governed by mass balance constraints in addition to demand constraints and feedstock availability. For heat integration between processes, the concept of process heat cascade integration is used. This approach allows heat exchange in the region between the pinch points of these processes, facilitating heat transfer integration and reducing energy consumption. The simultaneous solution is achieved by linking the mass and energy balance constraints through the calculation of the utility mass required by the processes. This eliminates the need for complex computational structures and iterative problem-solving, provided that all possible process combinations have been considered. The study evaluated the integration of different technologies to improve diversification and biofuel production in a sugarcane biorefinery. This sector is of great importance to the Brazilian economy and is considered essential for the sustainable development of a low-carbon economy. The results presented show that the integration of energy from the gasification process allowed the biorefinery to simultaneously generate revenue and energy from bagasse. Since it led to a significant improvement in the energy, economic, and environmental performance of the biorefinery, the production of methanol through bagasse gasification can be considered a key process for the expansion of the biorefinery. On the contrary, the conversion of carbon dioxide into methanol, while increasing the generation of carbon credits, has a significant negative impact on biorefinery energy efficiency and economic viability. This is due to the significant increase in electricity cost acquisition. The results also show that the price of electricity is critical to the economic viability of the biorefinery due to its high consumption of electrolyzers. Furthermore, the results indicate that the incorporation of the bagasse gasification process may be a viable technological alternative to conventional cogeneration systems. This is due to its demonstrated ability to meet the entire heat demand of the biorefinery.

## Figures and Tables

**Table 1:** Biorefineries technology cases.

Case	Route
1	Distillery + Photovoltaic Power Station (PPS)
2	Distillery + PPS + Vinasse Biodigestion (VBD)
3	Distillery+ PPS + VBD + Bagasse Gasification (BG)
4	Distillery+ PPS + VBD + BG + Methanol Catalytic Dehydration (MCD)
5	Distillery+ PPS + VBD + BG + Catalytic CO_2_ Hydrogenation (CCH)
6	Distillery+ PPS + VBD + BG + MCD + CCH

**Table 2:** Results of the optimal biorefineries configurations cost analysis.

Parameter	Case 1 ^1^	Case 2 ^2^	Case 3 ^3^	Case 4 ^4^	Case 5 ^5^	Case 6 ^6^
TAC [×10^6^ USD/year]	−91.23	−90.89	−113.85	−101.65	−48.76	−42.17
Total Investment [×10^6^ USD]	233.24	246.02	325.95	333.89	408.03	418.18
Resource bought [×10^6^ USD·y^−1^]	30.29	30.29	41.54	41.60	115.05	115.13
Biofuels Revenues [×10^6^ USD·y^−1^]	161.03	163.01	205.27	195.54	237.15	222.93
Carbon Credit Revenue [×10^6^ USD·y^−1^]	24.84	26.05	41.45	41.24	50.06	49.74
Labor Cost [×10^6^ USD·y^−1^]	57.37	60.52	80.18	82.13	100.37	102.87
Payback [y^−1^]	2.38	2.50	2.64	2.99	6.69	7.65

^1^ Destillery; ^2^ Destillery + Vinasse Biodigestion (VBD); ^3^ Destillery + VBD + Bagasse Gasification (BG); ^4^ Destillery + VBD + BG + Methanol Catalytic Dehydration (MCD); ^5^ Destillery + VBD + BG + Catalytic CO_2_ Hydrogenation (CCH); ^6^ Destillery + VBD + BG + MCD + CCH.

**Table 3:** Energy balance results of the optimal biorefinery cases.

Parameter	Case 1	Case 2	Case 3	Case 4	Case 5	Case 6
Energy Consumed [×10^9^ MJ·y^−1^]	11.71	11.71	12.36	12.36	16.76	16.76
Energy Produced [×10^9^ MJ·y^−1^]	5.88	6.256	9.21	9.33	11.14	11.31
Energy Efficiency [%]	50.25	53.43	74.49	75.43	66.46	67.46
Surface Power Density [GJ·ha^−1^]	171.17	182.00	253.74	257.12	288.83	293.44

## Data Availability

Data is contained within the article.

## Supplementary Materials

The following supporting information can be downloaded at https://www.mdpi.com/article/10.3390/e26060501/s1, Table S1: Main economic assumptions adopted for economic evaluation; Table S2. Values of CO_2_ emitted and avoided used to generate carbon credits. Table S3: Linearized cost curve coefficients and their respective levels; Table S4: Steam parameters considered; Table S5: Heat streams considered for each process; Table S6: IAR values used for each model considered in the cases; Table S7: OAR values used for each model considered in the cases.

## Nomenclature

Sets	
R	Resource set
U	Unit set
PU	Process subset
UT	Utilities subset
HUT	Hot utility subset
CUT	Cold utility subset
LRA	Subset for available resources
LRB	Subset for demanded resources
Subscripts	
u	Unit set index
r	Resource set index
pu	Process subset index
ut	Utility subset index
n	Stream index
l	Unit level index
Abbreviations and formulas	
CO_2_	Carbon Dioxide
CCH	Catalytic CO_2_ Hydrogenation
CH_4_	Methane
DME	Dimethyl Ether
HC	Heat Cascade
HI	Heat Integration
H_2_S	Hydrogen Sulfide
MCD	Methanol Catalytic Dehydration
MeOH	Methanol
PA	Pinch Analysis
TSI	Total Site Integration
Variables and Parameters	
a_u,l_	Angular coefficient of linearized segment l of unit u
AF	Annualization factor
avail_r_	Available amount of resource r
b_u,l_	Linear coefficient of linearized segment l of unit u
bought_r_	Amount bought of resource r
CapMin_u,l_	Minimum capacity of unit u in level l
CapMax_u,l_	Maximum capacity of unit u in level l
CarbVal	Value of carbon credit
CC	Carbon credit revenue
cons_u,r_	Consumption of resource r by unit u
Cu	Adjusted capital cost for unit u
C0_u_	Annualized capital cost at the reference scale for unit u
demand_r_	Amount demanded of resource r
fop	Hours of operation in a year
hs_ut_	Available heat per mass of cold utility ut
hv_ut_	Available heat per mass of hot utility ut
IAR_u,r_	Inlet flow rate of resource r in unit u
LC	Other cost
massUtility_pu,ut_	Mass of utility ut consumed by unit pu
MC	Maintenance cost
MCp_pu,n_	Thermal capacity of stream n of process pu
MP_r_	Market price of resource r
MER_pu_	Minimum energy requirement of hot utility ut
OAR_u,r_	Outlet flow rate of resource r in unit u
OC	Operational cost
PC_r_	Commercialization revenue of resource r
PC_r_	Revenue commercialization from product r
prod_u,r_	Production of resource r by unit u
Qc_pu,s_	Heat demanded by the cold streams in stage s by process pu
Qf_pu,s_	Inlet heat into unit pu and stage s
Qh_pu,s_	Heat available by the hot streams in stage s by process pu
Qin_pu_	Heat received from another unit by unit pu
Qout_pu_	Heat supplied to another unit by unit pu
Qu_pu,ut_	Heat consumed by unit pu of utility ut
Qs_pu,s_	Outlet heat into unit pu and stage s
RC_r_	Acquisition cost of resource r
ResCost_r_	Cost of resource r
se	Scaling exponent
sold_r_	Sold amount of resource r
TAC	Total annualized cost
Te_s_	Inlet temperature of stage s
Tin_pu,n_	Inlet temperature of stream n of unit pu
Tout_pu,n_	Outlet temperature of stream n of unit pu
Tpinch_pu_	Pinch temperature of unit pu
Ts_s_	Outlet temperature of stage s
UCC_u_	Capital cost of unit u
UF_pu_	Minimum energy requirement of cold utility ut
UTout_ut_	Outlet temperature of utility ut
y_u,l_	Binary variable that selects a unit u in a linearized segment l
w_u_	Scale adjustment variable of unit u
wl_u,l_	Local scaling adjustment variable of unit u in level l
