# Supplementary material for: Simultaneous Optimization and Integration of Multiple Process Heat Cascade and Site Utility Selection for the Design of a New Generation of Sugarcane Biorefinery
Source: Entropy (Basel). 2024 Jun 8;26(6):501. doi: 10.3390/e26060501 (PMC11202769; doi:10.3390/e26060501)
Supplement: Supplementary file 1 [file entropy-26-00501-s001.zip › entropy-2973220-supplementary.pdf]

# Simultaneous optimization and integration of multiple process heat cascade and site utility selection for the design of a new generation of sugarcane biorefinery with neutral carbon valorization

Victor Fernandes Garcia [1,*], Adriano Viana Ensinas2

[1] Center of Engineering, Modeling and Social Science Applied - Federal University of ABC, Santo André, Brazil; v.garcia@ufabc.edu.br

[2] Department of Engineering - Federal University of Lavras, Lavras, Brazil; adriano.ensinas@ufla.br

* Correspondence: V.F.G.: v.garcia@ufabc.edu.br; A.V.E.: adriano.ensinas@ufla.br

**Tabela S1.** Main economic assumptions adopted for economic evaluation.

| Parameter | Value | Unit |
|---|---|---|
| Available resources prices | | |
| Sugarcane | 10,60 | USD/ton |
| Water | 1,00 | USD/ton |
| Demanded resources prices | | |
| bioethanol | 781,12 | USD/ton |
| biomethanol | 539,00 | USD/ton |
| bioDME | 686,00 | USD/ton |
| BioMethane | 340,98 | USD/ton |
| Electricity | 57,69 | USD/MWh |

**Table S2.** Values of CO2 emitted and avoided used to generate carbon credits.

| Parameter | Value | Unit |
|---|---|---|
| Emitted $CO_2$ | | |
| Sugarcane | 0,0347 | kgCO2/ton |
| Avoided $CO_2$ | | |
| Bioethanol | 2,47 | ton $CO_2$/ton |
| Biomethanol | 0,56 | ton $CO_2$/ton |
| BioDME | 1,91 | ton $CO_2$/ton |
| Biomethane | 4,18 | ton $CO_2$/ton |
| Electricity | 0,118 | ton $CO_2$/MWh |

**Table S3.** Linearized cost curve coefficients and their respective levels.

| Unit | Level | CapMax | CapMin | capCostA | capCostB |
|---|---|---|---|---|---|
| Distillery | L1 | 0,6 | 0,1 | 8.803,70 | 2.128,80 |
| Distillery | L2 | 1,9 | 0,6 | 5.565,20 | 4.269,40 |
| Distillery | L3 | 4,5 | 1,9 | 3.788,50 | 7.725,80 |
| Vin2BioCH4 | L1 | 0,6 | 0,2 | 1100,5 | 181,15 |

| | | | | | |
|---|---|---|---|---|---|
| Vin2BioCH4 | L2 | 1,9 | 0,6 | 607,3 | 465,89 |
| Vin2BioCH4 | L3 | 4,5 | 1,9 | 410,41 | 854,11 |
| Bag2MeOH | L1 | 0,6 | 0,1 | 13.325,00 | 2.500,80 |
| Bag2MeOH | L2 | 1,9 | 0,6 | 7.669,00 | 6.083,00 |
| Bag2MeOH | L3 | 4,5 | 1,9 | 5.115,00 | 11.350,00 |
| MeOH2DME | L1 | 0,6 | 0,2 | 528,08 | 127,70 |
| MeOH2DME | L2 | 1,9 | 0,6 | 327,26 | 265,94 |
| MeOH2DME | L3 | 5 | 1,9 | 219,5 | 487,17 |
| Bag2EEL | L1 | 0,6 | 0,2 | 4.180 | 784 |
| Bag2EEL | L2 | 1,9 | 0,6 | 2.392 | 1.944 |
| Bag2EEL | L3 | 4,5 | 1,9 | 1.649 | 3.432 |
| H2AEL | L1 | 0,6 | 0,2 | 3.058 | 236 |
| H2AEL | L2 | 5 | 0,6 | 2.074 | 1.166 |
| H2PEM | L1 | 1 | 0,2 | 3.325 | 421 |
| H2PEM | L2 | 9 | 1 | 2.439 | 1.442 |
| Cogen9 | L1 | 0,6 | 0,2 | 2.605,05 | 488,93 |
| Cogen9 | L2 | 1,5 | 0,6 | 1.585,70 | 1.115,70 |
| Cogen9 | L3 | 5 | 1,5 | 1.029,50 | 2.103,40 |
| Cogen6 | L1 | 0,6 | 0,2 | 2.605,05 | 488,93 |
| Cogen6 | L2 | 1,5 | 0,6 | 1.585,70 | 1.115,70 |
| Cogen6 | L3 | 5 | 1,5 | 1.029,50 | 2.103,40 |
| Cogen2 | L1 | 0,6 | 0,2 | 2.605,05 | 488,93 |
| Cogen2 | L2 | 1,5 | 0,6 | 1.585,70 | 1.115,70 |
| Cogen2 | L3 | 5 | 1,5 | 1.029,50 | 2.103,40 |
| CO2MeOH | L1 | 0,6 | 0,01 | 2.553,60 | 552,20 |
| CO2MeOH | L2 | 1,9 | 0,6 | 1.538,10 | 1.179,90 |
| CO2MeOH | L3 | 4,5 | 1,9 | 1.039,40 | 2.163,10 |
| Solar Panel | L1 | 100 | 0,1 | 5.700,00 | 0 |
| Solar Panel | L2 | 500 | 100 | 5.700,00 | 0 |

Vin2BioCH4: Vinasse Biodigestion; Bag2MeOH: Bagasse Gasification; MeOH2DME: Methanol Catalytic Dehydratation; Bag2EEL: Bagasse Powerplant; H2AEL: Hydrogen production by water alkaline electrolysis; H2PEM: Hydrogen production by proton exchange membrane, Cogen9: Cogeneration system producing saturated steam at 9 bar and electricity; Cogen6: Cogeneration system producing saturated steam at 6.5 bar and electricity; Cogen2: Cogeneration system producing saturated steam at 2.2 bar and electricity; CO2MeOH: Catalytic CO2 Hydrogenation.

**Table S4.** Steam Parameters considered.

| UTILITIES | UTin | UTout |
|---|---|---|
| Steam9 | 448,55 | 448,45 |
| Steam6 | 431,95 | 431,85 |
| Steam2 | 396,6 | 396,5 |
| Refrigeration water | 308,15 | 313,15 |

**Table S5.** Heat streams considered for each process.

| Unit | Nstream | Tin | Tout | dH |
|---|---|---|---|---|
| Distillery | 1 | 403,15 | 305,15 | 41,00 |
| Distillery | 2 | 305,15 | 301,15 | 12,20 |

| | | | | |
|---|---|---|---|---|
| Distillery | 3 | 377,05 | 308,15 | 3,00 |
| Distillery | 4 | 382,45 | 308,15 | 37,20 |
| Distillery | 5 | 351,45 | 308,15 | 8,60 |
| Distillery | 6 | 383,15 | 308,15 | 8,40 |
| Distillery | 7 | 358,05 | 308,14 | 19,50 |
| Distillery | 8 | 354,85 | 354,84 | 26,40 |
| Distillery | 9 | 351,45 | 351,44 | 7,40 |
| Distillery | 10 | 298,15 | 323,15 | 4,40 |
| Distillery | 11 | 307,35 | 378,15 | 44,00 |
| Distillery | 12 | 371,25 | 388,15 | 2,70 |
| Distillery | 13 | 368,65 | 403,15 | 14,60 |
| Distillery | 14 | 304,35 | 363,15 | 33,70 |
| Distillery | 15 | 382,45 | 382,46 | 43,70 |
| Distillery | 16 | 287,05 | 377,06 | 21,80 |
| Distillery | 17 | 407,65 | 407,66 | 6,70 |
| Distillery | 18 | 422,75 | 422,76 | 2,50 |
| Bag2MeOH | 1 | 337,05 | 294 | 21,64 |
| Bag2MeOH | 2 | 368,65 | 371,95 | 25,35 |
| Bag2MeOH | 3 | 298,15 | 374 | 52,33 |
| Bag2MeOH | 4 | 495,55 | 333 | 100,14 |
| Bag2MeOH | 5 | 273,15 | 373 | 5,16 |
| Bag2MeOH | 6 | 423,15 | 423 | 52,90 |
| MeOH2DME | 1 | 305,15 | 417,15 | 4,52 |
| MeOH2DME | 2 | 417,15 | 428,15 | 9,93 |
| MeOH2DME | 3 | 428,15 | 523,15 | 2,03 |
| MeOH2DME | 4 | 645,15 | 443,15 | 4,76 |
| MeOH2DME | 5 | 320,15 | 318,95 | 12,60 |
| MeOH2DME | 6 | 425,55 | 428,65 | 1,86 |
| MeOH2DME | 7 | 337,55 | 329,65 | 7,69 |
| MeOH2DME | 8 | 363,35 | 369,75 | 6,09 |
| CO2MeOH | 1 | 410,15 | 318,15 | 1,44 |
| CO2MeOH | 2 | 430,15 | 318,15 | 1,80 |
| CO2MeOH | 3 | 431,15 | 318,15 | 1,96 |
| CO2MeOH | 4 | 530,15 | 308,15 | 7,16 |
| CO2MeOH | 5 | 364,15 | 493,15 | 24,11 |
| CO2MeOH | 6 | 508,15 | 313,15 | 66,66 |
| CO2MeOH | 7 | 390,15 | 308,15 | 14,53 |
| CO2MeOH | 8 | 393,35 | 390,65 | 17,70 |
| CO2MeOH | 9 | 426,05 | 433,95 | 37,41 |

Bag2MeOH: Bagasse Gasification; MeOH2DME: Methanol Catalytic Dehydratation; CO2MeOH: Catalytic $CO_2$ Hydrogenation.

**Table S6.** IAR values used for each model considered in the cases.

| UNITS↓ | Resources → CANA | Bag | EtOH | Vin | BioCH4 | H2 | MeOH | DME | EEL | Steam9 | Steam6 | Steam2 | ufW | VinTrat | H2O | CO2 |
|---|---|---|---|---|---|---|---|---|---|---|---|---|---|---|---|---|
| Distillery | 500 | 0 | 0 | 0 | 0 | 0 | 0 | 0 | 14 | 0 | 0 | 0 | 0 | 0 | 437,5 | 0 |
| Vin2BioCH4 | 0 | 0 | 0 | 438,48 | 0 | 0 | 0 | 0 | 1,026 | 0 | 0 | 0 | 0 | 0 | 0 | 0 |
| Bag2MeOH | 0 | 138,4 | 0 | 0 | 0 | 0 | 0 | 0 | 15,207 | 0 | 0 | 0 | 0 | 0 | 77,24 | 0 |
| MeOH2DME | 0 | 0 | 0 | 0 | 0 | 0 | 32,69 | 0 | 0,134 | 0 | 0 | 0 | 0 | 0 | 0 | 0 |
| H2AEL | 0 | 0 | 0 | 0 | 0 | 0 | 0 | 0 | 54 | 0 | 0 | 0 | 0 | 0 | 9 | 0 |
| H2PEM | 0 | 0 | 0 | 0 | 0 | 0 | 0 | 0 | 52,11 | 0 | 0 | 0 | 0 | 0 | 9 | 0 |
| Cogen9 | 0 | 142,38 | 0 | 0 | 0 | 0 | 0 | 0 | 0 | 0 | 0 | 0 | 0 | 0 | 0 | 0 |
| Cogen6 | 0 | 122,907 | 0 | 0 | 0 | 0 | 0 | 0 | 0 | 0 | 0 | 0 | 0 | 0 | 0 | 0 |
| Cogen2 | 0 | 94,79 | 0 | 0 | 0 | 0 | 0 | 0 | 0 | 0 | 0 | 0 | 0 | 0 | 0 | 0 |
| ufWFonte | 0 | 0 | 0 | 0 | 0 | 0 | 0 | 0 | 0,35 | 0 | 0 | 0 | 0 | 0 | 465,964 | 0 |
| CO2MeOH | 0 | 0 | 0 | 0 | 0 | 8,020 | 0 | 0 | 27,357 | 0 | 0 | 0 | 0 | 0 | 0 | 60,790 |

Vin2BioCH4: Vinasse Biodigestion; Bag2MeOH: Bagasse Gasification; MeOH2DME: Methanol Catalytic Dehydratation; Bag2EEL: Bagasse Powerplant; H2AEL: Hydrogen production by water alkaline electrolysis; H2PEM: Hydrogen production by proton exchange membrane, Cogen9: Cogeneration system producing saturated steam at 9 bar and electricity; Cogen6: Cogeneration system producing saturated steam at 6.5 bar and electricity; Cogen2: Cogeneration system producing saturated steam at 2.2 bar and electricity; CO2MeOH: Catalytic CO2 Hydrogenation. EtOH: Ethanol; Vin: Vinasse; BioCH4: Methane; H2: Hydrogen; MeOH: Methanol; DME: Dymethil Ether; Steam9: Saturated Steam at 9 bar; Steam6: Saturated Steam at 6.5 bar; Steam2: Saturated Steam at 2.2 bar; ufW: refrigeration water; VinTrat: Treated Vinasse.

**Table S7.** OAR values used for each model considered in the cases

| UNITS↓ | Resources → | | | | | | | | | | | | | | | |
|---|---|---|---|---|---|---|---|---|---|---|---|---|---|---|---|---|
| | Sugarcane | Bagasse | EtOH | Vin | BioCH4 | H2 | MeOH | DME | EEL | Steam9 | Steam6 | Steam2 | ufW | VinTrat | H2O | CO2 |
| Distillery | 0 | 138,24 | 32,4 | 447,84 | 0 | 0 | 0 | 0 | 0 | 0 | 0 | 0 | 0 | 0 | 0 | 30,500 |
| Vin2BioCH4 | 0 | 0 | 0 | 0 | 1,312 | 0 | 0 | 0 | 0 | 0 | 0 | 0 | 0 | 434,72 | 0 | 2,41 |
| Bag2MeOH | 0 | 0 | 0 | 0 | 0 | 0 | 39,78 | 0 | 0 | 0 | 0 | 0 | 0 | 0 | 0 | 0 |
| MeOH2DME | 0 | 0 | 0 | 0 | 0 | 0 | 0 | 23,179 | 0 | 0 | 0 | 0 | 0 | 0 | 0 | 0 |
| Bag2EEL | 0 | 0 | 0 | 0 | 0 | 0 | 0 | 0 | 30 | 0 | 0 | 0 | 0 | 0 | 0 | 0,000 |
| H2AEL | 0 | 0 | 0 | 0 | 0 | 1 | 0 | 0 | 0 | 0 | 0 | 0 | 0 | 0 | 0 | 0 |
| H2PEM | 0 | 0 | 0 | 0 | 0 | 1 | 0 | 0 | 0 | 0 | 0 | 0 | 0 | 0 | 0 | 0 |
| Cogen9 | 0 | 0 | 0 | 0 | 0 | 0 | 0 | 0 | 39 | 378,000 | 0 | 0 | 0 | 0 | 0 | 0,000 |
| Cogen6 | 0 | 0 | 0 | 0 | 0 | 0 | 0 | 0 | 39 | 0 | 308,66 | 0 | 0 | 0 | 0 | 0,000 |
| Cogen2 | 0 | 0 | 0 | 0 | 0 | 0 | 0 | 0 | 39 | 0 | 0 | 211,464 | 0 | 0 | 0 | 0,000 |
| ufWFonte | 0 | 0 | 0 | 0 | 0 | 0 | 0 | 0 | 0 | 0 | 0 | 0 | 2490,87 | 0 | 0 | 0 |
| CO2MeOH | 0 | 0 | 0 | 0 | 0 | 0 | 36,470 | 0 | 0 | 0 | 0 | 0 | 0 | 0 | 21,513 | 0 |

Vin2BioCH4: Vinasse Biodigestion; Bag2MeOH: Bagasse Gasification; MeOH2DME: Methanol Catalytic Dehydratation; Bag2EEL: Bagasse Powerplant; H2AEL: Hydrogen production by water alkaline electrolysis; H2PEM: Hydrogen production by proton exchange membrane, Cogen9: Cogeneration system producing saturated steam at 9 bar and electricity; Cogen6: Cogeneration system producing saturated steam at 6.5 bar and electricity; Cogen2: Cogeneration system producing saturated steam at 2.2 bar and electricity; CO2MeOH: Catalytic CO2 Hydrogenation. EtOH: Ethanol; Vin: Vinasse; BioCH4: Methane; H2: Hydrogen; MeOH: Methanol; DME: Dymethil Ether; Steam9: Saturated Steam at 9 bar; Steam6: Saturated Steam at 6.5 bar; Steam2: Saturated Steam at 2.2 bar; ufW: refrigeration water; VinTrat: Treated Vinasse.
